# Studies reporting bodily feedback effects on motivation have poor evidentiary value: a critical review and z-curve analysis

**DOI:** 10.1007/s00426-026-02382-7

**Published:** 2026-09-22

**Authors:** Shaheed Azaad

**Affiliations:** University of Muenster, Muenster, Germany

## Abstract

The embodied view of cognition holds that cognitive and emotional processes are grounded in our bodily experiences and physical interactions with the world. In line with this view, embodied motivation studies have purported to show that enacting motivation-related postures, expressions, and actions can elicit or modulate their corresponding motivational states. While theory-building reviews on the topic have accepted these studies’ conclusions at face value, a closer reading of key articles reveals several statistical issues that call their veracity into question. The present review shows that evidence for bodily feedback effects on motivation relies on: (1) studies using multiple measures of motivation, and dismissing results from those that do not support hypotheses, (2) analytical degrees of freedom inflating the false positive rate, and (3) reviews on the topic interpreting non-significant hypothesis tests as if they were significant. Supporting this analysis of the literature, a *z*-curve of 51 significant results reported across 18 articles indicated little evidentiary value, with an estimated replicability rate of just 0.21 (95% CI = [0.13, 0.25]). In sum, there is little credible evidence that bodily postures modulate motivational states.

The *embodied* view of cognition holds that cognitive and emotional processes are grounded in our bodily experiences and physical interactions with the world (Wilson, 2002). One hypothesis stemming from this view is that *bodily feedback* from postures, expressions and actions can elicit their corresponding emotional and cognitive processes. Early research on the topic claimed to find evidence for this hypothesis, for example, by showing that having participants “smile” by holding a pen in their mouth can increase feelings of amusement (Strack et al., 1988). However, while some of the seminal studies claiming to find phenomena in support of the embodied cognition hypotheses have since failed to replicate (Machery, 2024 for a review), other phenomena appear more robust (Coles et al., 2019, 2022). One phenomenon yet to be scrutinised is the effect of bodily feedback on *motivation* (Price & Harmon-Jones, 2016 for a review). The present study explores the evidentiary value of this literature by estimating its replicability using a *z*-curve analysis (Bartoš & Schimmack, 2022).

## Embodied cognition research and replicability

Work on the embodied cognition hypothesis has been the subject of considerable scrutiny following psychology’s replication crisis (Machery, 2024 for a review). Much of the seminal research on the topic was conducted at a time when studies were underpowered, questionable research practices were prevalent, and publication bias led to non-significant results being placed in the file drawer. Consequently, more recent work on the topic has sought to determine which, if any, of the embodied cognition phenomena reported in the literature are replicable. This endeavour has produced mixed findings, complicated by debates over what constitutes a valid replication (Harmon-Jones et al., 2025).

A notable example comes from replication projects focused on Strack et al.’s (1988) pen-in-the-mouth paradigm. A multi-lab replication by Wagenmakers et al. (2016) failed to replicate the effect on amusement across 17 labs that recruited over 1,800 participants. However, a subsequent study by Noah et al. (2018) argued that this was due to a methodological difference between the multi-lab replication and Strack et al.’s original study, namely that the replication project filmed participants. In their study, Noah et al. found that participants exhibited the pen-in-the-mouth effect when no video camera was present, but not when one was. However, not only was the difference between these conditions only marginally significant, but a subsequent multi-lab replication again failed to find the effect, this time with no camera present (Coles et al., 2022).

This does not mean, however, that there are no bodily feedback effects on emotion at all. Coles et al.’s multi-lab study (2022) did find that voluntarily held (versus covertly using a pen) smiles increased reported happiness. Likewise, a recent *z*-curve analysis has shown more robust evidence for embodiment in the domain of *body specificity* – specifically, the association of one’s dominant side with positive valence (Dapica et al., 2025). Given this heterogeneity in replicability, it is difficult to infer the replicability of one phenomenon from another, even if they share the same underlying theory. The present study focuses on bodily feedback effects on *motivation*. Generally, these studies report that high-approach (e.g., leaning forward) and low-approach (e.g., reclining) behaviours amplify and suppress approach motivation, respectively (Price & Harmon-Jones, 2016 for a review). Ideally, high-powered registered reports would tell us about the replicability of these effects. However, when such replications fail, they sometimes prompt post hoc explanations from the original authors, who attribute the result to deviations from the original paradigm that one might not expect to matter based on the theory (Sleegers et al., 2024).

However, according to a set of replication guidelines recently published by Harmon-Jones et al. (2025) in *Motivation Science*, replicating bodily feedback effects on motivation may require prohibitively high levels of precision. The authors detailed several factors that, if not controlled, could invalidate a replication effort. Some of these guidelines include: “Consider the physical environment on the way to the lab room” (p. 241), “Consider the physical environment of the lab room (e.g., cameras, mirror, room size, social setting)” (p. 241), and “Be mindful of the psychological state of participants at the start of the experiment” (p. 241). It is difficult to satisfy such guidelines for two reasons. One, because original studies do not report details about things like the size of the room in which they were conducted, much less the physical environment on the way to the lab room. Two, because it is difficult to determine what it means to “consider” or “be mindful (of)” these factors. For instance, it is not obvious how a researcher can determine what a participant’s “psychological state” *should* be at the beginning of the experiment for an effect to replicate. Holding these underspecified guidelines as a standard makes it difficult for researchers to conduct ‘valid’ replications and enables authors of original studies to make post hoc claims of improper methods when their study fails to replicate.

## Bodily feedback effects on motivation

Given that, as Harmon-Jones et al. (2025) argue, there is a plethora of factors that can invalidate a replication, the present study assesses how credible the original studies were in the first place. Reviews on bodily feedback effects on motivation make a compelling argument, citing a host of studies that apparently show that holding motivationally relevant postures can activate or modulate the corresponding motivational states (Harmon-Jones et al., 2014; Price & Harmon-Jones, 2016). A closer look at these studies, however, reveals that the evidence is less conclusive than reviews on the topic suggest.

Part of the reason this evidence is overstated is that researchers take multiple measures of approach motivation but place little weight on those that do not support their hypotheses. One example comes from Harmon-Jones and Peterson (2009), who presented participants with a stimulus designed to evoke anger (an approach-related emotion) while they were either reclined or upright. As the authors predicted, left frontal asymmetry (FA) in cortical activity – a measure of approach motivation – was greater in the upright condition. Although the authors concluded that the supine (low-approach) posture suppressed approach-motivational processes, it had no effect on participants’ self-reported anger. Harmon-Jones and Peterson state that FA and self-reported anger *can* differ independently, but they do not explain why the absence of an effect on anger should not be taken as a failure to support the bodily feedback hypothesis. This inconsistency, along with a recent meta-study that failed to find an association between approach motivation and FA (Paul et al., 2025), suggests that Harmon-Jones and Peterson’s FA effect might be better explained by processes unrelated to motivation.

Another example comes from Price et al. (2013), who asked participants to hold either a determined (high approach) or satisfied (low approach) facial expression. One measure of approach motivation in this study was how long participants persisted on a difficult task; this measure did not yield a significant effect. Rather than concluding that their hypothesis was not supported, the authors note that participants in their study held the expression for only one minute, compared to a previous study that asked participants to hold a posture for eight minutes (Riskind & Gotay, 1982), without explaining how this difference in duration should matter, or why facial expressions held for one minute apparently did produce an effect in their previous study (Price & Harmon-Jones, 2010).

Price et al. (2013) also administered a self-report measure of approach motivation, the PANAS-X (Positive and Negative Affect Scale; Watson et al., 1988). Specifically, they administered three of the positive subscales: interest, joy, and (positive) activation. Despite prior work from the same group suggesting that scores on the positive activation subscale partly reflect approach motivation (Harmon-Jones et al. 2009a, 2009b), Price et al. did not test whether activation differed between conditions; instead, they reported the mean values in a table. I conducted *t*-tests based on these summary statistics and found that the high approach condition did not produce significantly more positive activation than either the neutral (*p =*.734) or the low approach conditions (*p =*.623). Again, Price et al. did not interpret this as a failed test of the bodily feedback hypothesis.

Meanwhile, the FA effects that do appear to be significant might be better explained as artefacts of uncorrected multiple comparisons. Studies typically compute FA using data from one or more of three pairs of EEG electrodes – mid-frontal, frontal-central, and lateral-frontal. Analysing these sites separately inflates the Type I error rate, especially because one can also average across arbitrary combinations of them. How these data are analysed varies from study to study without an obvious reason. For example, Harmon-Jones and Peterson (2009) report data only from lateral-frontal electrodes and merely mention that “no other asymmetry indices differed between conditions” (p. 1210), treating data from mid-frontal and frontal-central sites as irrelevant to their hypothesis. In a later study, Harmon-Jones et al. (2011) analysed FA averaged across the three frontal sites. Price and Harmon-Jones (2011) differ again in their approach, reporting separate analyses for the three sites and finding significant effects at midfrontal and frontal-central sites but not at lateral-frontal sites.

Concerningly, studies that analyse FA at each site separately do not correct for multiple comparisons and treat their hypotheses as supported even when just one of the three sites yields the predicted effect (Price et al., 2013). The lack of multiple-comparison corrections, together with the fact that FA effects at any given site vary across studies, raises questions about the veracity of apparent postural effects on FA. It is no surprise that recent studies have failed to replicate these FA effects altogether (Harmon-Jones & Sun, 2021; Sun & Harmon-Jones, 2020).

Further, it is open to question whether some significant bodily feedback effects would remain if not for specific, inconsistent analytical decisions by researchers. Participants are usually asked to hold one of three postures/expressions corresponding to low, neutral, or high-approach motivation (Price et al., 2013; Price & Harmon-Jones, 2010). Rather than entering these data into a one-way ANOVA, some studies employ planned contrasts, which can boost statistical power by enabling one-tailed tests of directional hypotheses. The reasoning behind the structure of these planned comparisons, however, is opaque, and they vary seemingly arbitrarily across studies. For instance, whereas some studies contrast the neutral and low-approach conditions with the high approach condition (Price et al., 2013), others effectively omit the neutral condition altogether (Price & Harmon-Jones, 2011).

In Price et al. (2013), the authors applied different contrasts to different measures of motivation in the same experiment. Here, they compared the high approach condition to the neutral and low approach conditions in their FA data (i.e., + 2, −1, −1) but compared the high approach and neutral conditions to the low approach condition in their self-report measure of joy (i.e., −1, −1, + 2). The authors state that “statistical tests that were predicted, directional, based on theory, and specified in advance were evaluated with planned contrasts and a one-tailed criterion of significance (p. 223)” and that “according to the motivational direction model and other theoretical perspectives, satisfaction is low in approach motivation and thus should be associated with similar levels of left frontal cortical activity as neutral states (p. 223)”. But it is unclear why the authors made an a priori prediction that the pattern would differ between EEG and self-report measures of the same construct. Additionally, the authors do not explain why they expected low approach and neutral conditions to yield similar FA, even though they previously predicted and found effects using a (+ 1, 0, −1) contrast (Price & Harmon-Jones, 2011).

This inconsistency in contrasts suggests they are exploratory rather than truly planned, and because these studies were not preregistered, we cannot confirm that the contrasts were indeed determined a priori. It is also unclear why embodied motivation researchers include a neutral condition in their design at all if they plan to combine its data with one of the focal conditions (e.g., using +2, −1, −1 weights) or to effectively exclude it from analysis altogether (e.g., assigning it a weight of 0 in a linear contrast). If researchers are uninterested in data from the neutral condition, it would make more sense to remove it from the design and instead place participants in the low or high approach conditions to boost statistical power. Rather than serving a theoretical purpose, neutral conditions seem to instead afford an analytical degree of freedom that has likely inflated the literature’s Type I error rate. Moreover, if such contrasts are not truly determined a priori, the use of one-tailed tests might have contributed additional false positives (Hales, 2024).

Finally, some effects that reviews have marshalled in favour of the embodied motivation hypothesis are not statistically significant, yet they are presented as such. Specifically, this issue arises when researchers erroneously contrast significant and non-significant effects descriptively, even though the difference between them is not significant (Gelman & Stern, 2006; Nieuwenhuis et al., 2011). One instance of this comes from Price et al. (2012), who showed participants erotic and neutral images while they either leaned forward or reclined backward. The authors argued that leaning forward should amplify approach motivation toward erotic, but not neutral, images, as measured by startle eye-blink responses and late positive potentials (LPPs) in EEG data. Despite not finding the critical 2 × 2 interaction between posture and image type on either measure, the authors claim to have supported their hypothesis because the effect of reclining was significant for erotic but not neutral images^1^. Embodied motivation reviews (Harmon-Jones et al., 2014; Price & Harmon-Jones, 2016) have adopted a similarly optimistic interpretation of a study by Wiers et al. (2011), which examined whether an association task involving avoidance-related movements toward alcohol-related pictures could improve treatment outcomes for alcoholic inpatients. While these reviews have claimed that the intervention decreased alcohol cravings based on a significant pre-post *t*-test, the original paper reports that the critical 2 × 2 interaction, comparing the intervention and control groups, was not significant.

## The present z-curve

The Type I error-inflating practices described in this review were common prior to psychology’s credibility revolution, and many researchers engaged in them without realising their consequences. The lack of pre-registration, though unsurprising given the time period, casts doubt on whether measures, analyses, and data preprocessing were determined post hoc in ways that could increase the risk of false positives.

What we now know to be questionable research practices, along with publication bias toward significant results, has likely resulted in the evidence for bodily feedback effects on motivation being dramatically overstated. Given that most of the work in the field was conducted pre- credibility revolution (see Table 1), it is likely that issues surrounding credibility extend beyond the handful of studies I have reviewed here. Ironically, the studies described above are especially susceptible to critique because they are transparent about the different conditions and measures they include. It is possible that the identified issues extend to other studies on the topic that may not have been as transparent and therefore appear more credible on the surface.

In the present study, I conducted a *z*-curve analysis (Bartoš & Schimmack, 2022) to quantify the impact of the outlined issues on the replicability of studies reporting bodily feedback effects on motivation.

## Method

### The z-curve method

The *z*-curve method (Bartoš & Schimmack, 2022) involves assessing the distribution of significant *p*-values (transformed into *z*-scores) in a body of research. The distribution of *p*-values is informative because, while *p*-values are uniformly distributed under the null hypothesis, they depend on statistical power under the alternative hypothesis (Bartoš & Schimmack, 2022; Simonsohn et al., 2014). In terms of *z-*values, this would mean that, when the alternative hypothesis is true and studies are high-powered, *z*-values well above 1.96 should be more common than those only slightly above it. The z-curve method models the observed distribution of significant *z*-values as a mixture of studies with different levels of power, using that distribution to estimate the average power of a set of studies.

Obtaining the average power of a set of studies allows for two useful estimates. The first is the *expected discovery rate* (EDR), which reflects the estimated statistical power of included studies. An EDR substantially smaller than the observed discovery rate (ODR) indicates either publication bias or *p*-hacking. The second value is the *expected replication rate* (ERR), which estimates the proportion of significant results that would remain significant if the study were replicated exactly with the same sample size. Importantly, efforts to validate the *z*-curve method on real-world data from replication databases show that its EDR/ERR estimates correlate strongly with actual replication rates and may even overestimate ERR when true replicability is low (Bartoš & Schimmack, 2022; Röseler, 2023; Sotola, 2023).

### Search strategy

**Fig. 1:**
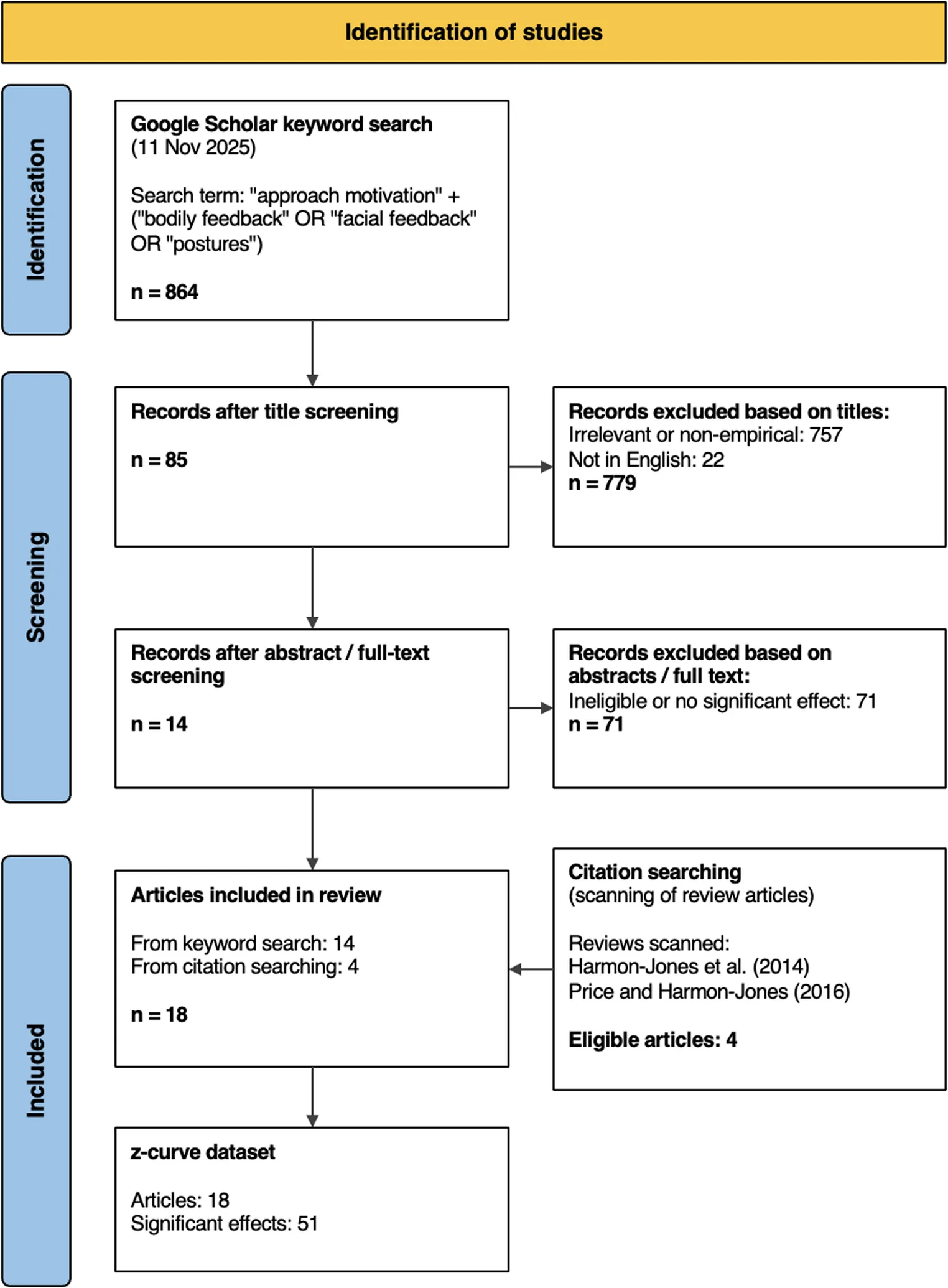
PRISMA literature search flowchart

The literature search involved two stages (Fig. 1). First, I conducted a Google Scholar keyword search through *Publish or Perish* software for macOS (Harzing, 2007). I used the search term *“approach motivation” + (“bodily feedback” OR “facial feedback” OR “postures”)* to capture empirical work investigating the effect of motivation-related postures, actions, and expressions on motivational states. The second stage involved scanning selected articles for other potentially relevant work. The scanned articles were two reviews on embodied motivation (Harmon-Jones et al., 2014; Price & Harmon-Jones, 2016).

### Inclusion criteria

Studies were eligible for inclusion if they reported at least one significant hypothesis test where:

1. Participants held either a motivation-related posture (e.g., reclining/leaning) or a facial expression of a motivation-related emotion (e.g., determination), or performed a motivation-related action (e.g., arm flexion).
2. This posture/expression/action affected a measure of motivation. These were typically direct effects, such as on left FA in cortical activity, self-reported emotions (e.g., anger), behaviours (e.g., consumption), or attitudes (e.g., desire to purchase). Indirect effects were also included (e.g., on rationalisations; Harmon-Jones et al., 2015), provided the authors hypothesised or concluded that the effect was mediated by differences in motivation. Studies investigating general positive or negative affect were not included. This is because positive and negative emotions do not always map onto approach and avoidance motivations, respectively. For example, anger is considered an approach-related emotion in the literature (Carver & Harmon-Jones, 2009).

I only included significant tests of the bodily feedback hypothesis. Although, as I noted in the introduction, some studies report non-significant tests, these are often not treated as (failed or inconclusive) hypothesis tests. Rather than making judgments about which measures should count as valid, which could be open to different interpretations and therefore debate, I chose to focus on those that authors in the field themselves took as evidence for bodily feedback effects. However, I did not include statistically invalid tests despite the authors’ interpretation. For example, simple main effects in the absence of a significant interaction (e.g. Price et al., 2012).

Inclusion of multiple effects from a single study was possible (e.g., from multiple measures), but not multiple statistics from the same data (e.g., an omnibus ANOVA and its post-hoc comparisons). In such cases, I included the statistic from the primary hypothesis test (e.g., the ANOVA) but not its post hoc comparisons.

The literature search yielded 51 eligible significant effects from 18 sources (Table 1)^2^. I detail the search process in the Supplemental Materials. Data and analysis scripts are available at https://osf.io/td4ur/overview?view_only=0f5ddf89331145c4b9e7ed1c65e3a047

### Sensitivity analysis

I ran simulations to assess the robustness of the present *z*-curve using the method described by Bartoš (2024). First, I examined robustness to decisions about which effects to include. This involved incrementally replacing a random subset of effects with those simulated from a high-powered (1- β = 0.80) study. Thus, when all effects are replaced, the *z*-curve should represent an unbiased set of studies. Of interest was whether replacing a small proportion of the effects would dramatically alter replicability estimates, suggesting low robustness of the present results. Second, I assessed how sensitive the present results were to potentially missing studies, for example, because the search criteria did not capture them. This involved adding, rather than replacing, simulated effects from a high-powered study. Again, of interest was the number of effects required to substantially change the *z*-curve’s credibility estimates. I simulated each replacement/addition 1000 times.

**Table 1:** z-curve disclosure table

Source	Study	Condition/DV	Effect description	Statistic	One sided	*p*
Harmon-Jones and Peterson (2009)	1		Planned contrast comparing FA in the insult-upright to neutral upright and insult-reclined conditions	t(38) = 2.70	no	0.010
Price and Harmon-Jones (2010)	1	Weak exemplars	Planned linear contrast comparing category fit ratings between reclining versus the remaining conditions	t(39) = 2.74	yes	0.005
Price and Harmon-Jones (2010)	2	Weak exemplars	Planned linear contrast comparing category fit ratings between the upright and reclining condition only	t(39) = 1.78	yes	0.041
Harmon-Jones et al. (2011)	1		2 × 2 interaction between posture and image type	F(1, 41) = 5.08	no	0.030
Price and Harmon-Jones (2011)	1	Not smiling; midfrontal sites	Planned linear contrast comparing FA between upright and reclining conditions	t(84) = 2.28	yes	0.013
Price and Harmon-Jones (2011)	1	Not smiling; frontal central sites	Planned linear contrast comparing FA between upright and reclining conditions	t(84) = 3.10	yes	0.001
Price et al. (2013)	1	Midfrontal sites	Planned contrast comparing FA in the determined vs. satisfied and neutral conditions	t(44) = 2.21	yes	0.016
Price et al. (2013)	1		Planned contrast comparing determination between determined and neutral conditions only	t(44) = 2.12	yes	0.020
Harmon-Jones et al. (2015)	1		2 × 2 interaction between posture and difficulty on goal ratings	F(1, 47) = 7.65	no	0.008
Harmon-Jones et al. (2015)	2	Difficult decision	2 × 2 × 2 interaction between posture, pre-postdecision, and chosen vs. rejected alternative	F(1, 42) = 8.60	no	0.005
Harmon-Jones and Sun (2021)	1	Participants with enough errors	Posture × correctness interaction on ERNs	F(1,24) = 13.72	no	0.001
Harmon-Jones and Sun (2021)	1	Participants who made errors on White-Gun trials	Posture × correctness interaction on ERNs	F(1,48) = 5.61	no	0.022
Sun and Harmon-Jones (2020)	1	CPz	Posture × congruence interaction on N450	F(1,34) = 7.65	no	0.009
Sun and Harmon-Jones (2020)	1	Pz	Posture × congruence interaction on N450	F(1,34) = 9.83	no	0.004
Krpan and Schnall (2014)	1		Motivation orientation × stimulus valence interaction on estimated distance	F(1, 73) = 4.72	no	0.033
Krpan and Schnall (2014)	2		Motivation orientation × stimulus valence interaction on estimated distance	F(2,74) = 30.67	no	< 0.001
Förster (2004)	2		Arm position × stimulus valence interaction on desire to buy product	F(2,81) = 5.26	no	0.007
Förster (2002)	1		Comparison of cookie consumption in arm flexion vs. extension conditions	t(18) = 2.34	no	0.031
Förster (2002)	2		3 × 2 interaction between posture and drink appetitiveness on drink consumption	F(2,90) = 5.92	no	0.004
Liu et al. (2022)	1		Effect of touch mode on willpower	F(1,116) = 4.30	no	0.040
Liu et al. (2022)	2		2 × 2 interaction between touch mode and health orientation	F(1,136) = 5.13	no	0.025
Liu et al. (2022)	3		Effect of interaction mode on adherence determination	F(2,138) = 111.3	no	< 0.001
Liu et al. (2022)	3		Effect of interaction mode on personal hygiene lapses	F(2,138) = 63.25	no	< 0.001
Miragall et al. (2021)	1		Effect of posture on motivation (from ANCOVA)	F(2,79) = 3.79	no	0.027
Koole et al. (2022)	1		Interaction between posture and trait anger on state anger	t(122) = 2.48	no	0.015
Koole et al. (2022)	2	State anger	Interaction between posture and trait anger	t(147) = 2.45	no	0.015
Koole et al. (2022)	2	Voodoo task	Interaction between posture and trait anger	chi(2,145) = 6.41	no	0.041
Koole et al. (2022)	3		Interaction between posture and trait anger on negative picture assignments	t(177) = 2.20	no	0.029
Koole et al. (2022)	4		Interaction between posture and trait anger on noise blasting	t(171) = 2.42	no	0.017
Koole et al. (2022)	4		Interaction between posture and BAS on noise blasting	t(173) = 3.12	no	0.002
Koole et al. (2022)	4		Interaction between posture and narcissism on noise blasting	t(173) = 2.23	no	0.027
Riskind and Gotay (1982)	1		Posture effect on task persistence	F(1,16) = 7.73	no	0.013
Riskind and Gotay (1982)	2		Posture effect on task persistence	t(18) = 2.03	yes	0.029
Riskind and Gotay (1982)	3		Interaction between posture and Hammen-Krantz depression scale on mean perception scores	F(1,26) = 16.82	no	< 0.001
Harmon-Jones (2006)	1	Mid-frontal, central, parietal regions	MANOVA comparison of left- and right-hand contractions on approach affect	F(5,22) = 2.74	no	0.045
Harmon-Jones (2006)	1	Mid-frontal	Effect of hand contraction on FA	F(1,26) = 9.32	no	0.005
Harmon-Jones (2006)	1	Anterior temporal	Effect of hand contraction on FA	F(1,26) = 4.57	no	0.042
Harmon-Jones (2006)	1	Central	Effect of hand contraction on FA	F(1,26) = 10.19	no	0.004
Harmon-Jones (2006)	1	Parietal	Effect of hand contraction on FA	F(1,25) = 8.70	no	0.007
Peterson et al. (2008)	1	Noise loudness	Effect of hand contraction on aggression	t(22) = 1.8	yes	0.043
Peterson et al. (2008)	1	Mid-frontal	Effect of hand contraction on FA	t(22) = 1.85	yes	0.039
Peterson et al. (2008)	1	Lateral-frontal	Effect of hand contraction on FA	t(22) = 2.13	yes	0.022
Peterson et al. (2008)	1	Central	Effect of hand contraction on FA	t(22) = 3.57	yes	< 0.001
Peterson et al. (2008)	1	Frontal-temporal	Effect of hand contraction on FA	t(22) = 1.90	yes	0.035
Peterson et al. (2008)	1	Central-parietal	Effect of hand contraction on FA	t(22) = 2.93	yes	0.004
Peterson et al. (2008)	1	Anterior temporal	Effect of hand contraction on FA	t(22) = 2.28	yes	0.016
Van den Bergh et al. (2011)	1B		Product type × shopping instrument interaction	F(1,29) = 7.12	no	0.012
Van den Bergh et al. (2011)	2 A		Flexion × product type interaction	t(20) = 2.57	no	0.018
Van den Bergh et al. (2011)	2B		Flexion × temporal discounting interaction	t(52) = 2.43	no	0.019
Van den Bergh et al. (2011)	3		Interaction between muscle contraction and sensitivity to reward	F(1,95) = 4.50	no	0.036
Van den Bergh et al. (2011)	4	Dominant hand	Interaction between muscle contraction and sensitivity to reward	F(1,31) = 8.13	no	0.008

## Results

**Fig. 2:**
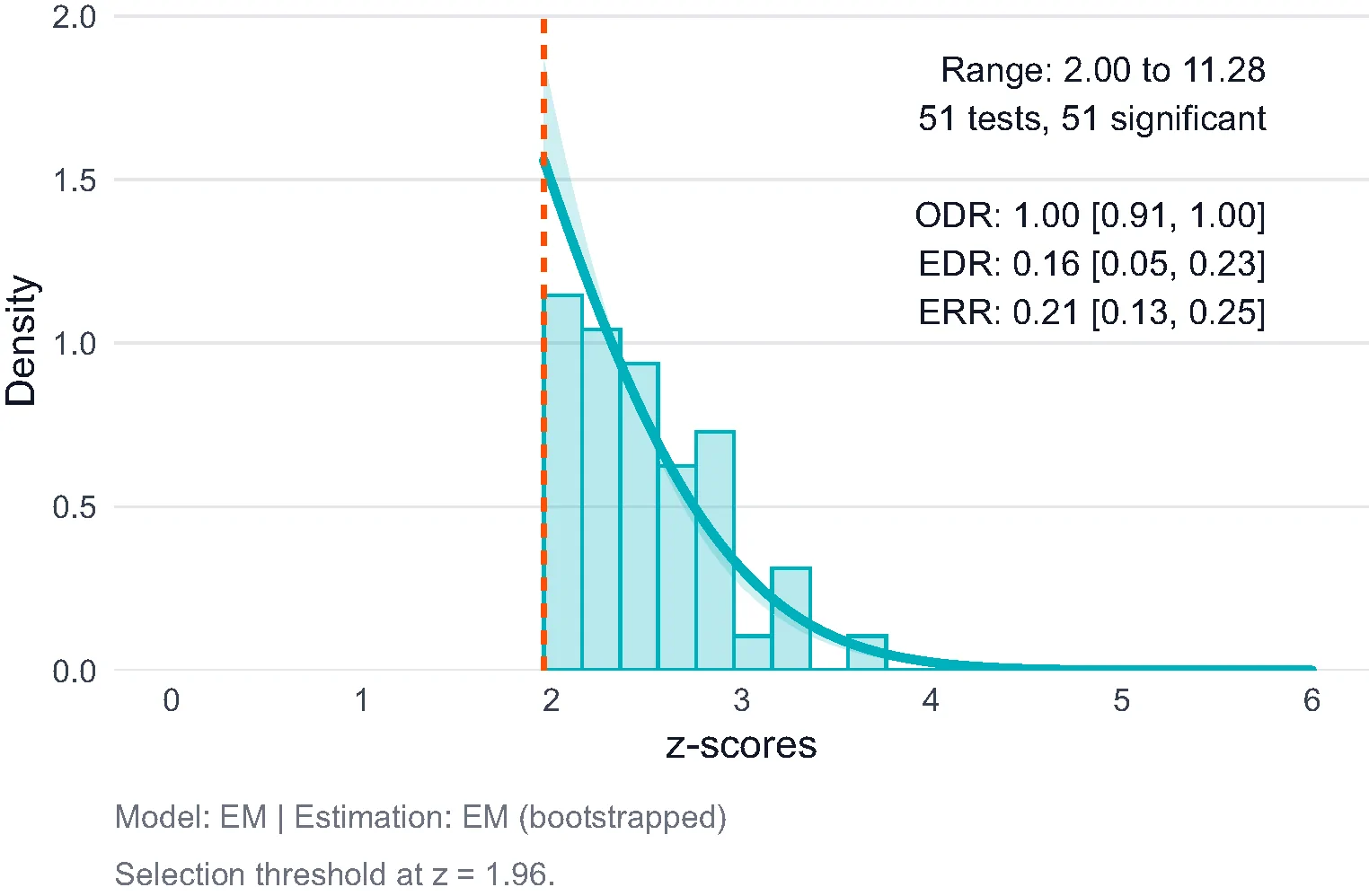
z-curve of significant bodily feedback effects on motivation

To prepare data for *z*-curve analysis, I computed precise *p*-values from reported *t-* and *F-*statistics using the *zcurve* package (Bartoš & Schimmack, 2020) for *R*. This produced two-tailed *p*-values. To ensure that *p-*values matched those reported in the literature, I divided them by 2 when the authors reported a one-tailed test. Because some experiments reported multiple significant effects from the same sample, there were dependencies between *p*-values in the dataset. I accounted for this by fitting a clustered *z-*curve (using bootstrapping), with effects clustered by sample (Fig. 2). This *z*-curve analysis indicated that the included studies had low evidential value, yielding an ERR of just 0.21 (95% CI [0.13, 0.25]). The ODR, which is 1.00 (95% CI [0.91, 1.00]) here since I only extracted significant effects, was substantially larger than the EDR of 0.16 (95% CI [0.05, 0.23]) with no overlap in confidence intervals. Notably, the lower bound of the 95% CI is 0.05, so the possibility that the true EDR is simply the Type I error rate cannot be ruled out.

As an anonymous reviewer commented, the *z*-curve package also produces two additional estimates relevant to credibility. One is the file drawer ratio, which estimates how many non-significant tests might not have been published. In this case, the file drawer ratio is uninformative because I excluded published non-significant effects; thus, the absence of such effects could not be attributed to publication bias. Another value of interest is Sorić’s (1989) maximum false discovery rate, which estimates the maximum proportion of tests that could be false positives. The maximum false discovery rate was estimated at 0.28 (95% CI [0.18, 1.00]). While this point estimate would suggest that over 70% of significant tests are of true alternative hypotheses, the upper bound of the 95% CI being 1.00 means that we cannot rule out the possibility that *all* significant effects analysed are false discoveries.

### Sensitivity analyses

**Fig. 3:**
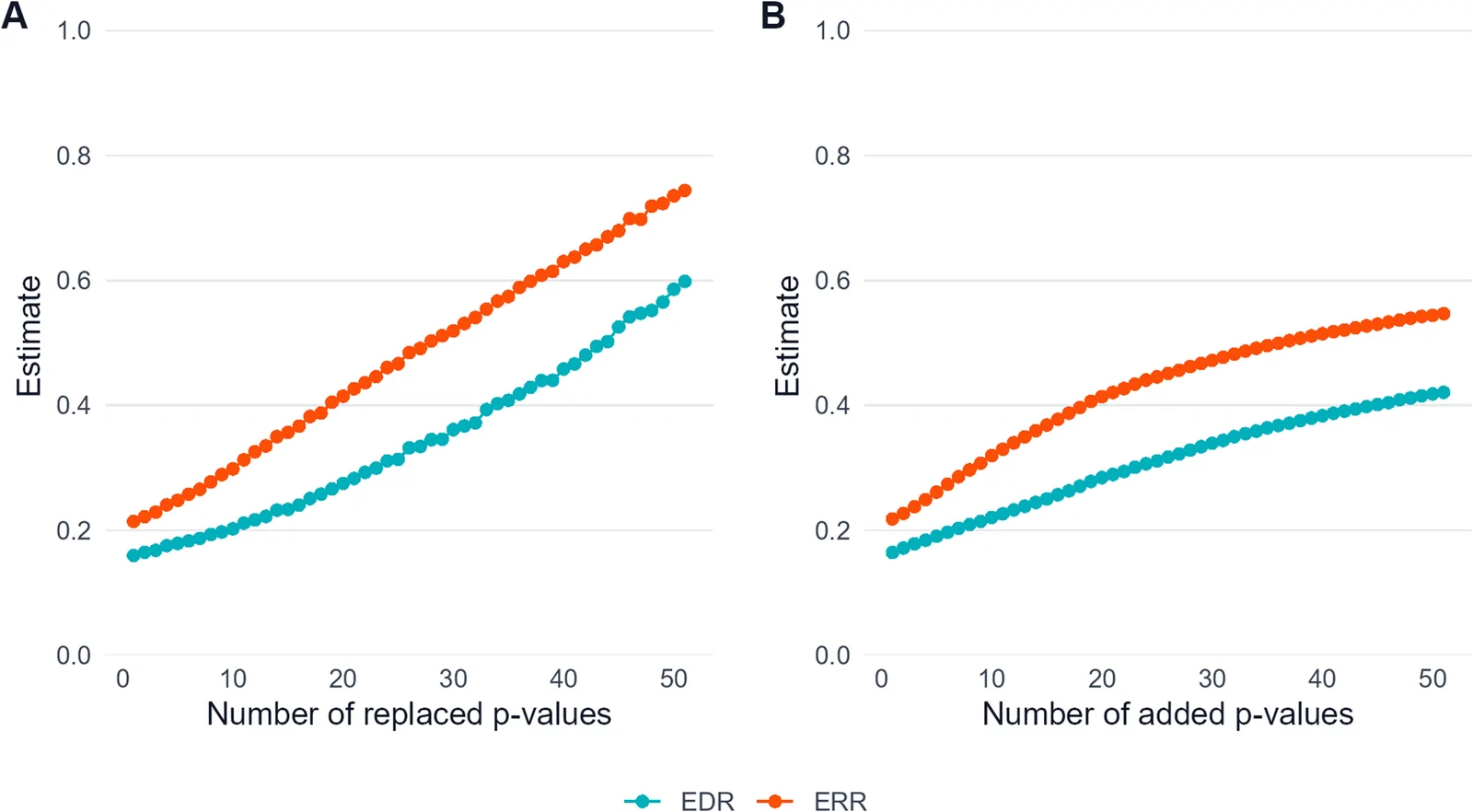
z-curve sensitivity to effect selection (**A**) and study inclusion (**B**)

Sensitivity plots (Fig. 3) suggest that the low credibility indicated by the present *z*-curve is unlikely to be an artifact of study coding or study inclusion criteria. ERR and EDR were around and under 0.50, respectively, even after replacing half of the included effects with simulated *p*-values (Fig. 3A). Additionally, even after doubling the number of effects in the present dataset, both ERR and EDR remained below 0.60 (Fig. 3B).

**Fig. 4:**
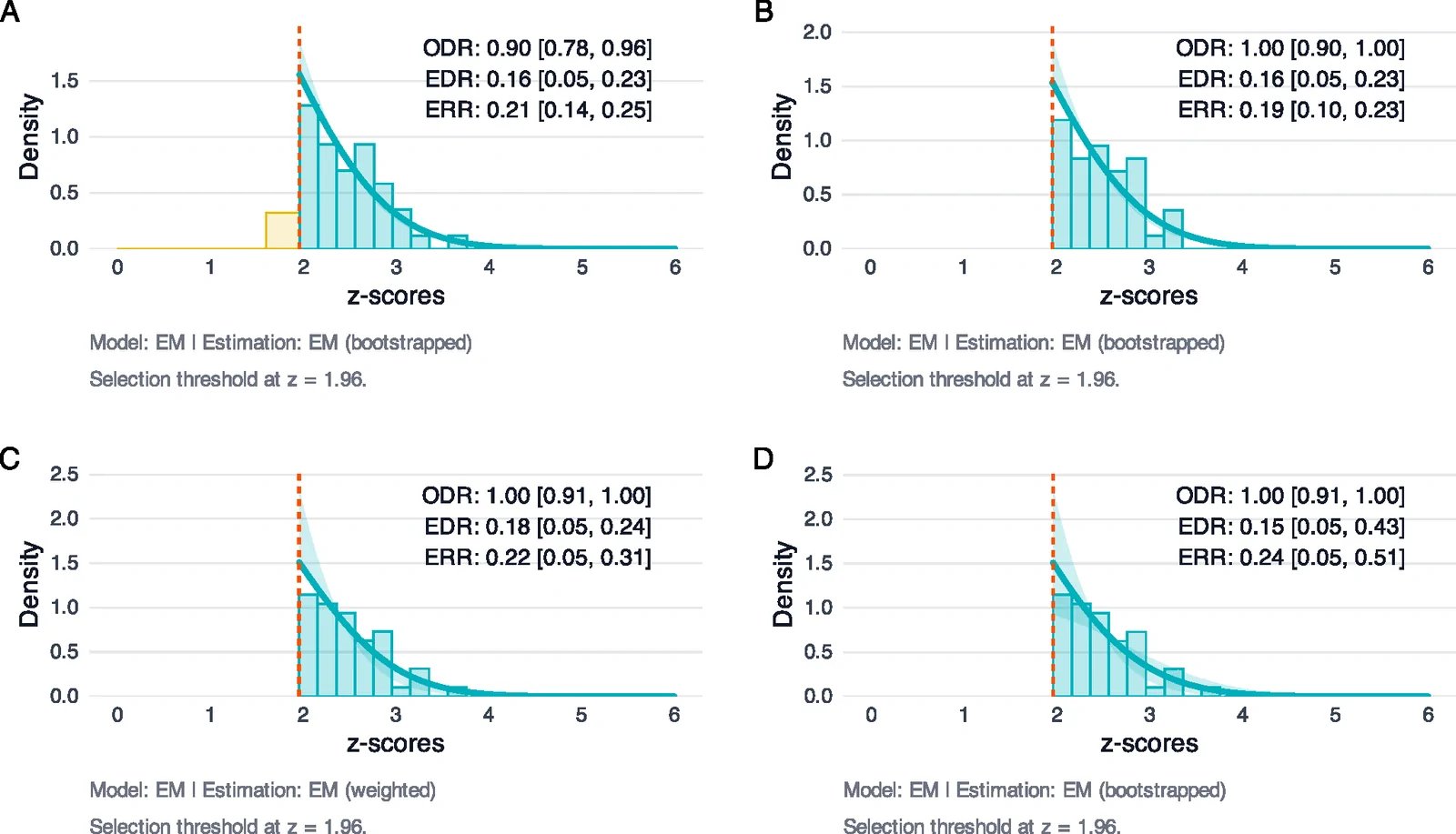
*z*-curves when (**A**) not halving *p*-values for reported one-tailed *t*-tests, (**B**) excluding edge case effects, (**C**) weighting effects by article, and (**D**) clustering by article rather than sample

In addition to decisions about study inclusion, several analytical decisions could have affected the results. I fit a series of *z*-curves to examine the impact of these decisions. Specifically, I fit an additional four *z*-curves: (1) Without halving *p*-values for one-tailed *t-*tests (Fig. 4A). (2) Excluding effects from two studies that used potentially edge-case motivation-related operationalisations (Fig. 4B). One of these studies defined pressing (versus tapping) as an approach-related behaviour (Liu et al., 2023), while the other treated narcissism as an approach-related trait (Koole et al., 2022). (3) Clustering effects using bootstrapping rather than weighting (Fig. 4C), and (4) Clustering effects by article rather than sample (Fig. 4D). Estimates of EDR and ERR did not differ substantially from the main *z-*curve.

## Discussion

Results confirmed what a critical reading of the literature already suggests – that there is little evidentiary value in studies purporting to show bodily feedback effects on motivation. Rather, it seems that the apparent support for these effects relies on ignoring non-significant findings, on statistical decisions that have inflated the literature’s Type I error rate, and on optimistic interpretations of marginally or non-significant effects. Even ignoring statistical issues, the present study’s findings are unsurprising, given how much of the literature relies on postural effects on FA, which have recently failed to replicate (Harmon-Jones & Sun, 2021; Sun & Harmon-Jones, 2020) and have not been supported by data from explicit and behavioural measures of approach motivation (Price et al., 2013).

As an anonymous reviewer pointed out, there are two reasons why, despite the results of the present *z*-curve, we cannot yet rule out the possibility of bodily feedback effects on motivation. The first reason is that the low ERR in the present study does not necessarily mean that there is *no* effect, but rather that there may be one that the *z*-curved studies were typically too underpowered to detect. For this reason, the field should move toward running high-powered, pre-registered replications of the studies central to the theory. But first, theoretical work is needed to articulate which predictions researchers should make. As discussed in the introduction, replications are limited in their ability to advance our understanding if they are explained away post hoc when they fail (Sleegers et al., 2024). For this reason, it needs to be established a priori which measures, contrasts, and manipulations must yield bodily feedback effects for the theory to be supported.

The second is that the ERR could reflect a combination of highly replicable and non-replicable studies. It could be, for example, that some measures or manipulations reliably yield bodily feedback effects on motivation, while others do not. The proportion of such studies would be quite small, especially given that the ERR of 0.21 is likely an *over*estimate of real-world replicability (Röseler, 2023). Currently, the lack of direct replications and pre-registered studies makes it difficult to determine which, if any, subset of studies might be robust. It does appear unlikely, though, that there is any reliable effect of bodily feedback on FA, given that (1) the highest-powered studies examining this effect have failed to replicate it (Harmon-Jones & Harmon-Jones, 2019; Sun & Harmon-Jones, 2020) and (2) meta-analyses suggest that there is little-to-no relation between FA and motivation once accounting for publication bias (Garrison et al., 2022; Kuper et al., 2019).

### Is motivation embodied?

Bodily feedback effects are a key prediction of the embodied motivation hypothesis. Results of the present *z*-curve raise questions about how we should interpret evidence of the reverse mechanism– motivational states priming associated actions (see Laham et al., 2014 for a meta-analysis). Although these effects appear more stable (Laham et al., 2014) and have been replicated in high-power, preregistered studies (Eder et al., 2021), embodiment implies a bidirectional relationship for which the evidence is currently poor.

One explanation might come from Eder et al. (2021), who used virtual reality stimuli to disentangle approach/avoidance actions from their outcomes. That is, an approach movement, like leaning forward, could bring stimuli closer, and vice versa. The study found that, in a virtual reality environment, unpleasant stimuli (a spider) primed whole-body movements away from them, whereas pleasant stimuli (a flower) primed movements toward them. However, this pattern was not reversed when the relationship between movements and their consequences was reversed. That is, unpleasant and pleasant stimuli still primed avoidance and approach behaviours, respectively, even when avoidance behaviours brought stimuli closer (in the virtual environment), and approach behaviours moved them further. The authors propose that such whole-body movements might have an overlearned association with approach/avoidance behaviours. In light of the present study’s results, it is possible that action priming effects are better explained in terms of stimulus-response compatibility (Simon et al., 1981) – i.e., in terms of conceptual overlap between stimulus and response properties – than embodiment.

### Implications for replication efforts in motivation and emotion research

Estimating replicability with the *z-*curve method sidesteps some of the hurdles associated with replicating original studies. As Harmon-Jones et al. (2025) argue, many factors can render a replication effort invalid. However, many of the critical details one would need to control for in a valid replication were not reported in the original studies. For instance, Harmon-Jones et al. suggest that researchers should “track emotion and personality variables of experimenters” (p. 241). Without knowing what these variables were in the *z*-curved studies, it is impossible to replicate them in this aspect. Furthermore, it is unclear what the acceptable ranges for these variables are, since the theory does not specify the boundary conditions for bodily feedback effects.

Convincing replication efforts are already disproportionately resource-intensive compared with the original studies they seek to replicate. Multi-lab replications have far higher power than original studies and require coordination among research groups. Placing null findings in a file drawer or *p*-hacking requires far less effort. Underspecified theory and replication guidelines exacerbate this problem, as they enable post hoc explanations that necessitate further replications (Sleegers et al., 2024). Concerningly, these guidelines are applied asymmetrically. For example, none of the *z*-curved studies report tracking the emotion and personality variables of experimenters as suggested by Harmon-Jones et al. (2025). The question then arises: why are replications, but not original studies, considered invalid if they do not exercise this level of control? Given the effort required to ‘debunk’ a false positive effect via replication studies, it may be worthwhile, as I have done here, to begin with critical reviews of original studies paired with a *z*-curve to estimate their replicability.

Moreover, until work is done to assess the credibility of a body of literature, it is perhaps best to treat surprising and novel findings with scepticism until directly replicated, rather than accepting them until they fail to replicate. Harmon-Jones et al. (2025) suggest that conceptual replications may be more valuable than direct replications, as they assess the generalisability of a phenomenon using different measures or paradigms. However, conceptual replications also allow researchers to explain away results that do not support their hypotheses. Specifically, if a conceptual replication finds the predicted effect, then researchers can call it a successful replication; if not, they can attribute the absence of an effect to differences in the design or measures. We see this flexibility in the embodied motivation literature – authors pay little attention to results from behavioural and explicit measures of motivation when they do not support their hypotheses, even though such results should be treated as failed replications (e.g., Price et al., 2013).

### Limitations and future directions

An anonymous reviewer of a previous version of this manuscript argued that studies were included arbitrarily. The first example the reviewer gave was of Krpan and Schnall (2014), who used perceived distance to stimuli as a measure of motivation. While the reviewer argued that this is “a measure of perception”, the authors themselves hypothesised that approach motivation should make stimuli appear closer. The second example came from Harmon-Jones and Sun (2021), where the dependent variable was error-related negativity (ERN) in EEG data. Here, the reviewer argued that ERN is a measure of conflict processing. While this is true, Harmon-Jones and Sun hypothesised that the postural effect on ERNs in their study – which is the effect I included – was mediated by a difference in motivation, arguing that “… a supine posture may reduce approach motivation generally and thus reduce the approach action-oriented (motivational) tendencies associated with the cognitions involved in generating the cognitive conflict or cognitive dissonance.” (p. 376). In both cases, the authors hypothesised that differences in their measure across conditions reflected differences in motivational orientation. Because my goal was to evaluate the credibility of the evidence put forth in the literature, I did not make decisions about whether the authors’ operationalisations were appropriate. In any case, the sensitivity analyses suggest that excluding these studies would have made little difference to the results.

The same reviewer also argued that I had improperly excluded relevant studies. One example they gave was from Krieglmeyer et al. (2010), which examined how stimulus valence facilitated motivationally relevant actions. This phenomenon cannot be attributed to bodily feedback because it examines how emotions can elicit postures/actions, rather than how postures/actions can elicit emotions. Another example that the reviewer gave was a study by Rothermund (2003), which did not manipulate body posture but examined how task feedback (success/failure) affected participants’ processing of valenced words. While other excluded studies may have been more relevant to the present *z*-curve, these examples highlight the challenges of falsifying or critiquing underspecified theory. After all, it is not obvious how one could, based on the current theory, derive inclusion criteria that would both exclude postural effects on motivation-related dependent variables (e.g., Krpan & Schnall, 2014) and include effects where posture was not manipulated at all (Rothermund, 2003).

Nevertheless, it is true that my search strategy may not have captured all studies investigating the effects of bodily feedback on motivation. For example, studies on consumption or purchasing behaviour that are not explicitly linked to motivation might not have appeared in the keyword search or been referenced in the scanned articles. As the sensitivity analyses showed, however, ERR would have remained below 0.60 even in the extremely unlikely case that (1) the present *z*-curve somehow only included half of the bodily feedback effects on motivation in the literature (i.e., ERR was low even after adding 51 simulated effects), and (2) this missing literature was several times more credible than the studies included here. There is no reason to believe that the present search strategy or inclusion criteria could have systematically omitted high-replicability studies. Importantly, because I scanned reviews for citations, the present z-curve at the very least indicates that the foundational research that informed major theoretical work (Harmon-Jones et al., 2014; Price & Harmon-Jones, 2016) in the field has low credibility.

One limitation of the present z-curve is that it focuses only on significant effects. This does not affect the key ERR estimate, which is based only on significant tests (Schimmack & Soto, 2026). The difference between the EDR and ODR, however, does depend on effect selection and, in this case, tells us little about publication bias. We can nevertheless conclude two things in the present study: (1) given the average power of the significant tests reported in the literature, far fewer of them should be significant (i.e., ODR vs. EDR), (2) we would expect very few of these significant effects in direct replications (i.e., ERR).

Future work should investigate whether other hypotheses in motivation research that have been tested in a similar way might also have less support than reviews on the topic suggest. For example, one of the studies in the present *z*-curve (Harmon-Jones & Sun, 2021) has been taken as evidence for the action-based model of dissonance (Harmon-Jones et al. 2009a, 2009b). Other work on this topic also has characteristics that may have inflated their Type I error rate, like failing to correct for multiple EEG comparisons (Harmon-Jones et al., 2008).

## Conclusion

In sum, there is little credible evidence that bodily postures modulate motivational states. Even though motivational priming effects appear more reliable (Laham et al., 2014), the lack of credible evidence for a bidirectional relationship between motivational states and their associated postures nevertheless casts doubt on the embodied view of motivation. Future work might consider running high-powered registered replications of the foundational work in the field. However, precisely what constitutes a valid replication of such studies must be determined a priori.

## Data Availability

Data and analysis scripts are available at https://osf.io/td4ur/overview?view_only=0f5ddf89331145c4b9e7ed1c65e3a047.

## Footnotes

1 In lieu of an interaction effect, the authors present a significant planned contrast comparing the neutral and erotic conditions, with different weights assigned to leaning-erotic and neutral-erotic conditions. This, of course, does not tell us whether the effect of posture differed between stimulus types. The authors argue that 2 × 2 ANOVAs are “most likely to yield a p <.05 when the means form an X pattern” (p. 214). But this does not mean that such a test is inappropriate, as Price & Harmon-Jones (2016) seem to argue in a later review, stating that “one of the problems with (traditional omnibus interaction tests) is that often they do not reveal meaningful comparisons between conditions” (p. 97). Rather, this is simply a matter of ordinal interactions typically being smaller than crossover interactions, which thus require adequate power to detect (Lakens & Caldwell, 2021).

2 There has been some debate about the validity of z-curves fitted on fewer than 300 effects. This debate has occurred via blog posts, but concluded with the author of the z-curve showing that EDR and ERR estimates are valid with as few as 50 effects, as long as there are at least 12 *z* values between 1.96 and 2.96 (Schimmack, 2026). The present dataset contains 40.
